# A novel ensemble learning method for crop leaf disease recognition

**DOI:** 10.3389/fpls.2023.1280671

**Published:** 2024-01-08

**Authors:** Yun He, Guangchuan Zhang, Quan Gao

**Affiliations:** ^1^ School of Big Data, Yunnan Agricultural University, Kunming, China; ^2^ Key Laboratory for Crop Production and Intelligent Agriculture of Yunnan Province, Yunnan Agricultural University, Kunming, China; ^3^ School of Mechanical and Electrical Engineering, Yunnan Agricultural University, Kunming, China

**Keywords:** crop disease, recognition, ensemble learning, weight, feature extraction performance

## Abstract

Deep learning models have been widely applied in the field of crop disease recognition. There are various types of crops and diseases, each potentially possessing distinct and effective features. This brings a great challenge to the generalization performance of recognition models and makes it very difficult to build a unified model capable of achieving optimal recognition performance on all kinds of crops and diseases. In order to solve this problem, we have proposed a novel ensemble learning method for crop leaf disease recognition (named ELCDR). Unlike the traditional voting strategy of ensemble learning, ELCDR assigns different weights to the models based on their feature extraction performance during ensemble learning. In ELCDR, the models’ feature extraction performance is measured by the distribution of the feature vectors of the training set. If a model could distinguish more feature differences between different categories, then it receives a higher weight during ensemble learning. We conducted experiments on the disease images of four kinds of crops. The experimental results show that in comparison to the optimal single model recognition method, ELCDR improves by as much as 1.5 (apple), 0.88 (corn), 2.25 (grape), and 1.5 (rice) percentage points in accuracy. Compared with the voting strategy of ensemble learning, ELCDR improves by as much as 1.75 (apple), 1.25 (corn), 0.75 (grape), and 7 (rice) percentage points in accuracy in each case. Additionally, ELCDR also has improvements on precision, recall, and F1 measure metrics. These experiments provide evidence of the effectiveness of ELCDR in the realm of crop leaf disease recognition.

## 1 Introduction

Crops face continuous threats from different diseases during planting, making disease control a long-standing and crucial challenge for farmers. Early detection of crop diseases is an essential task in agriculture (Applalanaidu and Kumaravelan, 2021). In the early stage, farmers and experts relied on their knowledge and experience to diagnose crop diseases. However, this approach is inefficient, expensive, and characterized by low accuracy. With the development of information technology, researchers began to apply machine learning (ML) and deep learning (DL) technologies for crop disease recognition. ML and DL technologies offer the potential to automate and enhance the accuracy of crop disease recognition. In recent years, DL has become the mainstream technology in the field of crop disease recognition due to its automated feature extraction, high accuracy, and user-friendliness. Many researchers tried to apply more advanced DL models to recognize diseases in different crops. For instance, Fuentes et al. (2018) used faster R-CNN to recognize tomato diseases, achieving a recognition rate of approximately 96%. Nachtigall et al. (2016) proposed a technique for apple disease recognition based on AlexNet, achieving an accuracy of 96.6%. Jiang et al. (2020) applied convolution neural networks (CNNs) to recognize four different rice diseases, achieving an accuracy of 96.8%. There are also many recognition studies on crops based on DL models, including grape (Xie et al., 2020), mango (Singh et al., 2019), millet (Coulibaly et al., 2019), olive (Cruz et al., 2017), and cucumber (Kawasaki et al., 2015). The DL models used in these studies include Xception, MCNN, VGG, LeNet, and custom CNN. In these studies, different DL models are applied by the researchers who expect that better models will bring better recognition accuracy.

There are many kinds of crops and crop diseases. While the leaves of different crops may exhibit distinct morphological features, the symptoms caused by different diseases can often appear visually similar (Ngugi et al., 2021). This brings a great challenge to the generalization performance of the recognition model. Different DL models have their own feature extraction mechanisms, resulting in different recognition performances in different crops’ disease recognition. This variability also makes it difficult to build a common model that can achieve optimal recognition performance across all crop types and diseases. To solve this problem, we proposed a novel ensemble learning method for crop leaf disease recognition (named ELCDR), which can integrate different DL models to improve the generalization performance of the recognition method.

The contributions of this paper include the following key aspects:

1) We proposed a novel ensemble learning method for crop leaf disease recognition (named ELCDR). Compared with the crop disease recognition methods that are based on a single model, ELCDR demonstrates better recognition performance and generalization performance.
2) We proposed an innovative ensemble learning strategy and deployed it within ELCDR. Compared with the traditional voting strategy, our strategy can realize more reasonable ensemble learning. By this, ELCDR can achieve better recognition performance and generalization performance than the methods based on the voting strategy.
3) We executed experiments on the dataset that includes four different crop types, and the results showed the effectiveness of our methods.

## 2 Related works

The recognition of crop leaf disease images essentially constitutes an image classification. In the past, many researchers tried to achieve automated crop disease image recognition using traditional machine learning technologies. Support vector machine (SVM) is the most widely used machine learning algorithm in the research field of crop disease image recognition. Raza et al (Shan-E-Ahmed et al., 2015). proposed an SVM-based method that can detect tomato powdery mildew with an accuracy of more than 90%. Islam et al. (2017) suggested an SVM-based approach for recognizing two potato diseases, with an accuracy of 95%. Additionally, Pantazi et al. (2019) proposed a multiple crop disease recognition system based on SVM, also achieving an accuracy of 95%. On the other hand, Kaur et al. (2018) applied SVM to recognize various diseases of soybean, with the highest accuracy reaching 62.53%. Furthermore, the k-nearest neighbor (KNN) (Hossain et al., 2019), k-means (Prakash et al., 2017), transductive support vector machine (TSVM) (Ahmed et al., 2019), and multiple linear regression (MLR) (Sun et al., 2018) are also the traditional machine learning technologies that are widely applied in crop leaf disease recognition. All of these recognition methods based on traditional machine learning need to select image features manually or by using other selection algorithms. The quality of feature selection significantly impacts the performance of recognition. This leads the traditional machine learning methods to have a certain threshold for use and may have an unstable performance.

In recent years, due to developments in deep learning technologies, researchers have been incorporating deep learning models into the realm of crop disease image recognition. Deep learning models are proficient at automating feature selection and extraction, allowing for end-to-end deployment. This has led deep learning models to gradually become the mainstream methods in the field of crop disease image recognition. For instance, Jiang et al. (2019) achieved real-time disease recognition for apples using the VGG model and attained an accuracy of 78.8%. Additionally, some studies have applied VGG to recognize other crops (Paymode and Malode, 2021) (Bhagat et al., 2023) (Kundu et al., 2021). VGG is a widely applied deep learning model in crop disease recognition studies because it has a simple network structure and a smaller convolutional kernel. Researchers also introduced other deep learning models to crop leaf disease recognition, such as ResNet (Stephen et al., 2023), MobileNet (Chen et al., 2021), AlexNet (Chen et al., 2022), and GoogLeNet (Yang et al., 2023). However, the diverse array of crops and diseases poses a great challenge to the generalization performance of recognition models. This challenge makes it very difficult to build a unified model that can achieve optimal recognition performance on all kinds of crops and diseases. When applying deep learning models to a new kind of crop or disease, researchers often need to optimize the model to adapt to the unique characteristics of that specific crop and disease (Ganesan and Chinnappan, 2022) (Reddy et al., 2023). Otherwise, the models may fail to achieve their optimal recognition performance.

In order to improve the generalization performance of the recognition method, researchers have introduced ensemble learning to image-based crop disease recognition (Li et al., 2021). Ensemble learning (Ganaie et al., 2022) is an effective way to improve the generalization performance of the recognition method. The authors of refs (Chaudhary et al., 2020) (Mathew et al., 2022) (Palanisamy and Sanjana, 2023). introduced the voting strategy of ensemble learning to crop disease recognition and observed improvements in recognition accuracy in the experiments. When we apply the voting strategy to recognize an image, each of the models costs a vote for a particular category. Then, the image is assigned to the category which receives most of the votes. Voting is the simplest and most effective ensemble learning strategy, but it treats all models as equally important. Even if a model fails to extract effective features, its vote still has significant weight. This is obviously unreasonable. Furthermore, if each model votes for a different category, it becomes challenging to determine which category should the image belong to, rendering the voting strategy ineffective.

**Figure 1:**
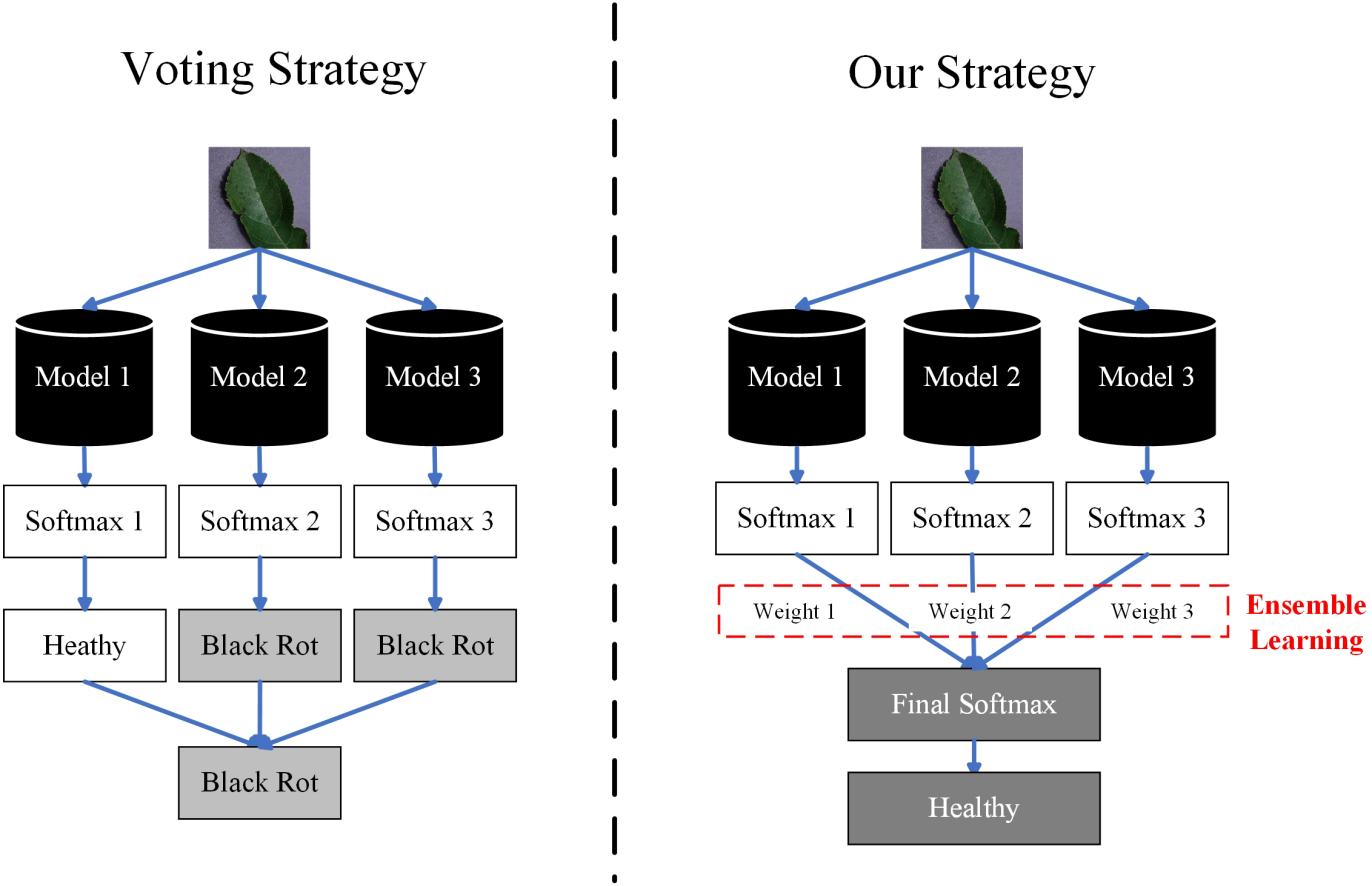
Comparison of the voting strategy and our strategy.

To solve this problem, we have proposed a novel ensemble learning method for crop leaf disease recognition method, named ELCDR. Different from the traditional voting strategy, it assigns varying weights to the models during ensemble learning. These weights are determined based on the feature extraction performance of each model, which can be measured by examining the distribution of feature vectors. Using this approach, ELCDR can achieve more accurate and stable disease recognition performance across different corps. **Figure 1** shows the primary differences between the voting strategy and our proposed strategy when applied in ensemble learning.

## 3 A novel ensemble learning method for crop leaf disease recognition

**Figure 2:**
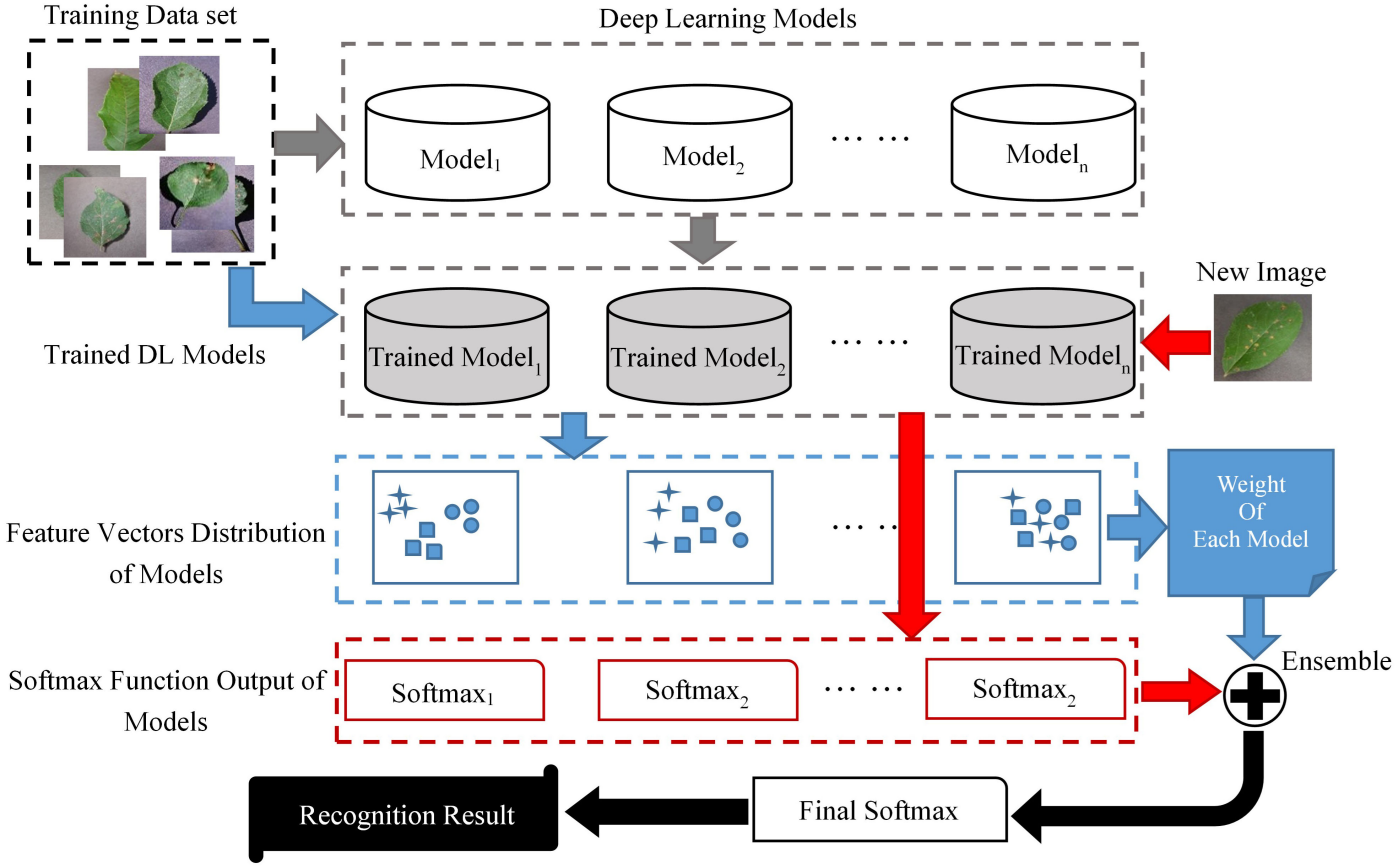
The basic flow of ELCDR.

The basic flow of ELCDR is shown in **Figure 1**. There are four stages in ELCDR, and they are represented by arrows of different colors in **Figure 2**.

1) Stage 1 is represented by the gray arrows. We build a training set and multiple DL models at the beginning of this stage. Then, we use the training set to train each of the DL models, respectively. If there are *n* DL models, then we will get *n* trained models at the last of this stage.

2) Stage 2 is represented by the blue arrows. This stage’s main purpose is to measure each model’s feature extraction performance, and then calculate the ensemble learning weight of each model. We input the training set into each of the trained models to get the feature vector distribution of each trained model, respectively. Then, we can get each model’s feature extraction performance by measuring the vector distribution of all the trained models. At last, we calculate the ensemble learning weight for each model based on their feature extraction performance. The details about how to measure the feature extraction performance of models are introduced in Section 3.1, and the details about how to calculate the ensemble learning weight are also introduced in Section 3.1. If there are *n* trained models, then we will get their ensemble learning weight as *ω*_1_ to *ω_n_* by this stage.

3) Stage 3 is represented by the red arrows. Once a new image is input into the trained models, we can get the softmax function output of each model. If there are *n* trained models, then we can extract their softmax function output as *sf*_1_ to *sf_n_*.

4) Stage 4 is represented by the black arrows. Based on the ensemble learning weight and the image’s softmax function output, we can calculate the final softmax function output by the following formula:


(1) $$sf_{final}=\sum_{i=1}^{n}\omega_{i}*sf_{i}.$$

In Formula (1), *n* means the number of trained models. Lastly, we can get the new image’s recognition result based on the final softmax function output. The details regarding how to calculate the final softmax function output are provided in Section 3.2.

### 3.1 Weight calculation by measuring the feature extraction performance of the models

**Figure 3:**
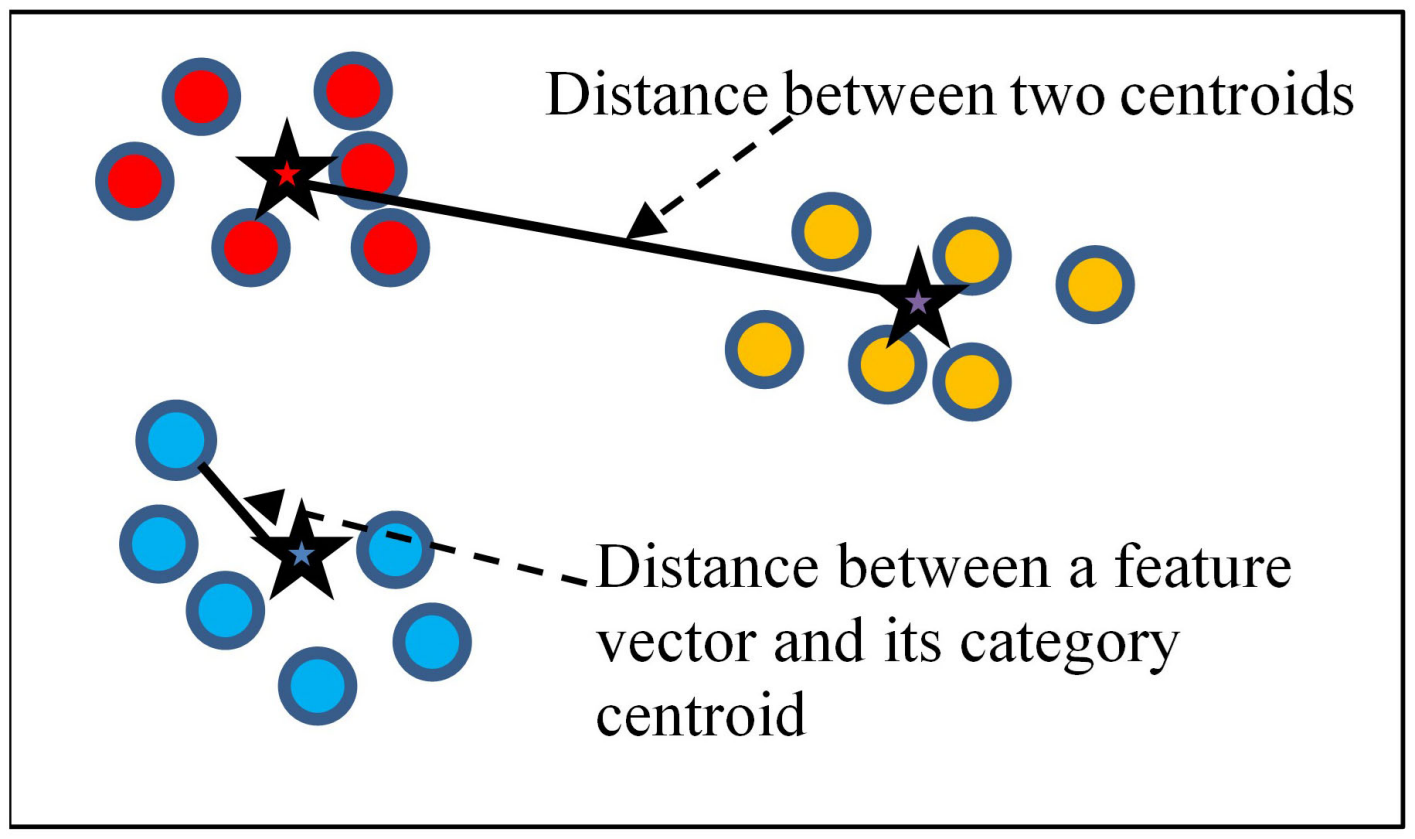
Example of feature vector distribution in a two-dimensional space.


The traditional ensemble learning method generally uses a voting weighting strategy, but we find this strategy to be irrational. Different DL models may exhibit different feature extraction performances on the same dataset. If a model could extract a more effective feature, it should be assigned more weight during the ensemble process. Otherwise, the model with less effective feature extraction should be assigned less weight. By this thought, we have introduced a novel weight calculation method that measures the feature extraction performance of the models. This method uses the vectors’ distribution of the training set to measure a model’s feature extraction performance. If there are *t* images in the training set, they will be divided into *k* categories. Once we input the training set into the DL model, we can get *t* feature vectors corresponding to the images. **Figure 3** shows an example of feature vector distribution in a two-dimensional space. In this space, there are 18 feature vectors represented by spots. All of the vectors can be divided into three categories, which we use different colors to represent. Each category has a category centroid represented by a star. The category centroid is an average vector of all vectors in the category. For a specific *category_p_*, if there are total *m* vectors in *category_p_*, its centroid *cen_p_* can be calculated by the following formula:

(2) $$cen_{p}=\sum_{i=1}^{m}vector_{i}^{\;p}.$$

We believe that a model’s feature extraction performance can be measured by considering the in-category distance and the between-categories distance of the training set feature vectors.

The in-category distance in a vector space means the average distance of all vectors to their respective category centroid. We use icD to represent the in-category distance, and icD can be calculated using the following formula:


(3) $$icD=\sum_{p=1}^{k}\sum_{i=1}^{m}\;f_{ED}\left(cen_{p},vector_{i}^{\;p}\right)/t.$$

In Formula (3), *k* means the total number of categories, and the function $f_{ED}\left(vector_{x},vector_{y}\right)$ means the Euclidean distance between two vectors.

The between-categories distance means the average distance between all category centroids. We use bcD to represent the in-category distance, and bcD can be calculated using the following formula:


(4) $$bcD=\;\sum_{p=1}^{k}\sum_{q=p+1}^{k}\;f_{ED}\left(cen_{p},cen_{q}\right)/C_{k}^{2}.$$

In Formula (4), $C_{k}^{2}$ is the combination number formula.

**Figure 4:**
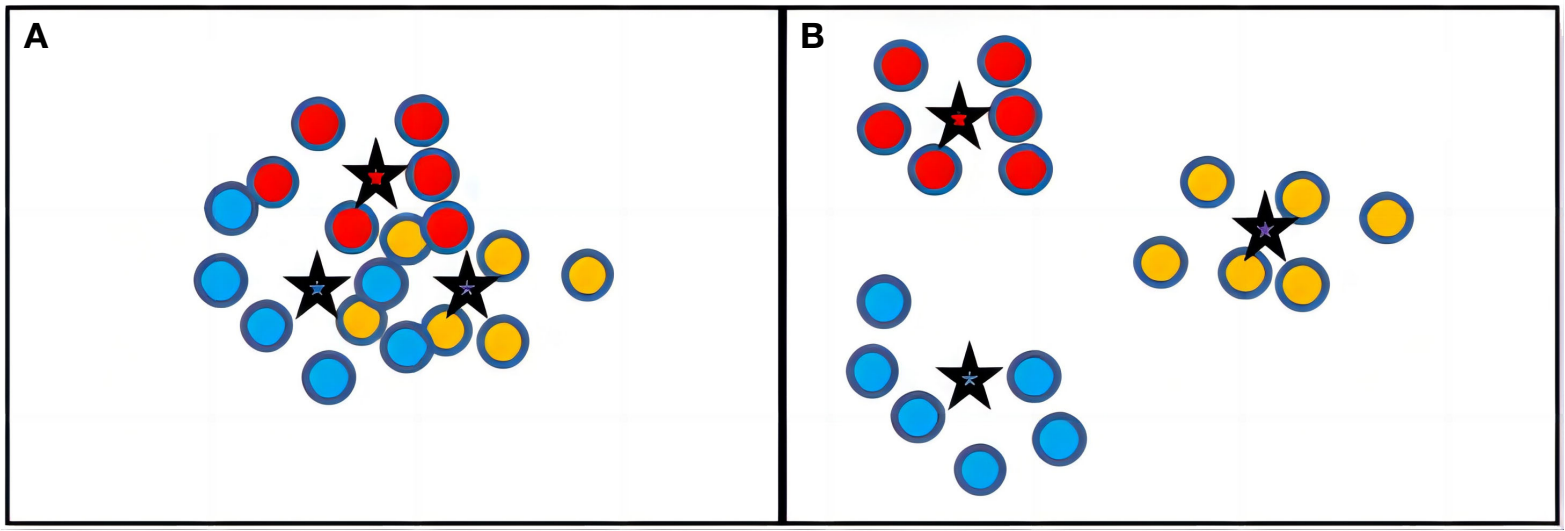
Example of feature vector distribution of different models. **(A)** Feature Vectors Distribution of *Model_A_*. **(B)** Feature Vectors Distribution of *Model_B_*.

If a model could effectively extract the images’ feature, then the feature vectors that belong to the same category should be closely distributed around their category centroid. Additionally, the category centroid of different categories should be distributed far apart from each other. As shown in **Figure 4**, there are two different models’ feature vector spaces (*Model_A_* and *Model_B_*). *Model_B_* obviously has evidently extracted a more effective feature because it is very easy to determine the category of an image in the vector space of *Model_B_*. In contrast, the vectors of *Model_A_* are closely distributed together, making it very challenging to distinguish the category of an image. Therefore, we can conclude that *Model_B_* has a higher feature extraction performance than *Model_A_*. From this perspective, we should assign a higher weight to *Model_B_* than to *Model_A_* during ensemble learning.

The distribution of vectors in the same category can be measured by icD , and the distribution of different categories can be measured by bcD . So, if a model has a bigger bcD and a smaller icD , then we could consume it to have better feature extraction performance. We use *FEP_g_* to represent the feature extraction performance of *model_g_*, and it can be calculated by the following formula:


(5) FEPg= bcDgicDg.

We should give the model that has a higher *FEP* more weight when performing ensemble learning. So, the weight formula is defined as follows:


(6) $$w_{g}=\frac{FEP_{g}}{\sum_{i=1}^{n}FEP_{i}}\;.$$

In this formula, $w_{g}$ means the weight assigned to *model_g_*, and *n* means the total number of models used during ensemble learning.

In ELCDR’s stage 2, we input the training set into the trained models and then calculate their weights by measuring their feature extraction performance.

### 3.2 Ensemble learning strategy of ELCDR

Once a new image is input to the trained DL models, we can get a softmax function output from each model. The softmax function output is the probability distribution of this image belonging to each category. For example, if we input *img_x_* into a trained DL model, we get a softmax function output as [0.2, 0.6, 0.2]. It means that there are three categories in total, and *img_x_* has the most probability that belongs to the second category. If we input the *img_x_* to multiple trained DL models, we would get the corresponding multiple softmax function output. Then, we can use the weighting strategy in Section 3.2 to integrate them into a final softmax function output $sf_{final}$ . $sf_{final}$ . is calculated as follows:


(7) $$sf_{final}=\sum_{i=1}^{n}w_{i}*sf_{i}.$$

In this formula, $sf_{i}$ means the softmax output of the *i*_th_ model, $\omega_{i}$ means the weight of the *i*_th_ model, and there are *n* models in total to ensemble learning. At last, we choose the category that has the most probability in the $sf_{final}$ as the final crop disease recognition result.

### 3.3 The basic steps of ELCDR

**Figure 5:**
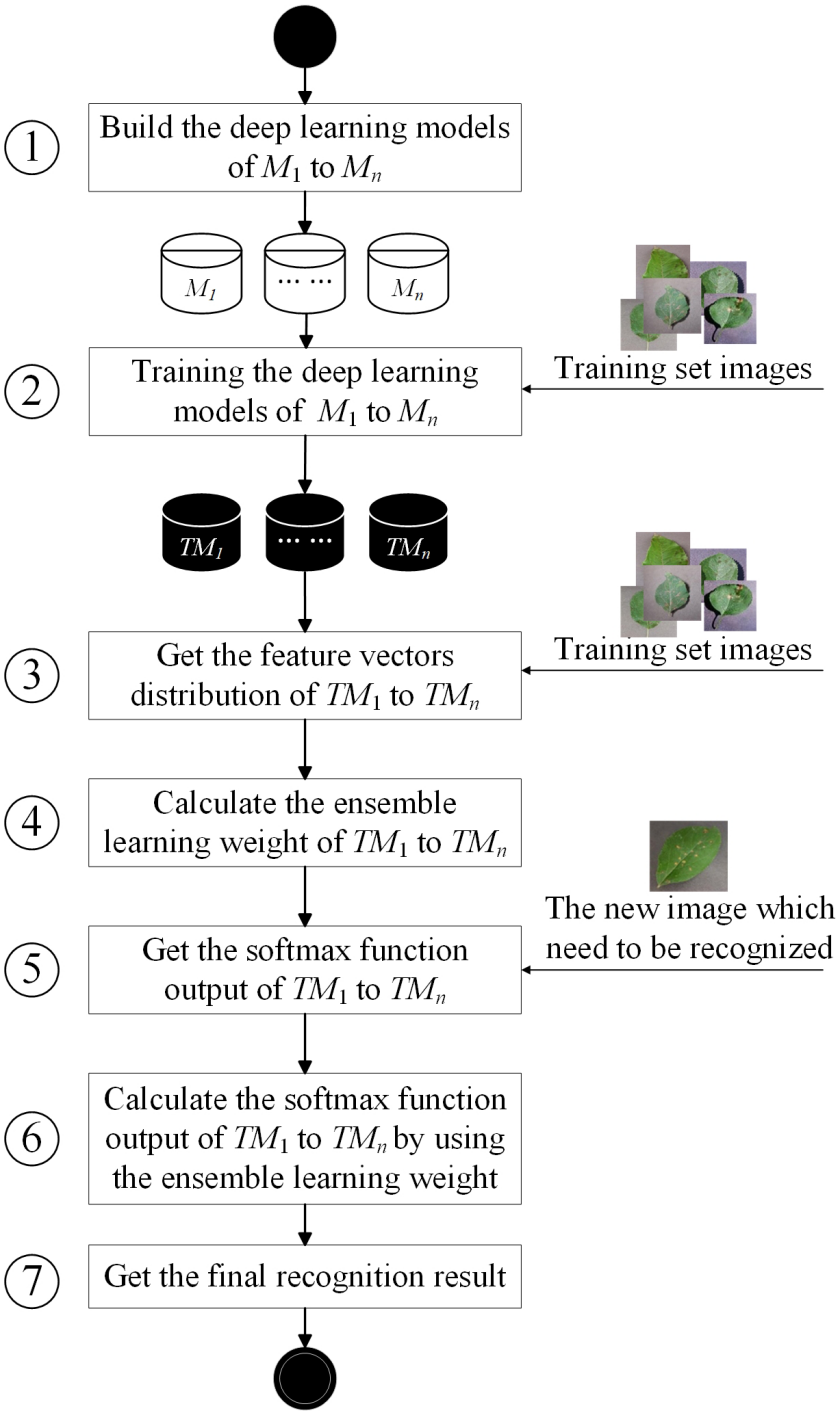
Basic flowchart of ELCDR.

The basic flowchart of ELCDR is shown in **Figure 5**, comprising seven basic steps. In this section, we will introduce the details of each step.

Step 1. In this step, we need to decide how many and which deep learning models for deployment in ensemble learning are necessary. If we aim to deploy *n* models, then we need to build these models and set their hyperparameters. Generally, we can choose the models that are widely used in crop disease recognition to deploy, such as VGG, ResNet, and MobileNet. After completing this step, we can have *n* models: *M*_1_, *M*_2_,…, and *M_n_*.

Step 2. After the step of building the model, we can use the training set images to train the models one by one. Each image in the training set is labeled to indicate its crop disease category. If there are *h* images in the training set, they could be divided into *k* crop disease categories. This means that for each *image_i_* ∈ {*image*_1_, *image*_2_,…, *image_h_*}, it will belong to a specific *category_j_* ∈ {*category*_1_, *category*_2_,…, *category_k_*}. After the training process, we can get *n* trained models: *TM*_1_, *TM*_2_,…, and *TM_n_*.

Step 3. In this step, we need to input the training set images to the trained models one by one and extract the feature vectors in the pooling layer of the trained models. For each *image_i_* ∈ {*image*_1_, *image*_2_,…, *image_h_*}, if we input it to *TM_g_*, then we could extract its feature vector in the pooling layer of *TM_g_*. Since there are *h* images, we can get *h* feature vectors in the feature vector space of *TM_g_*. Then, we can calculate the feature extraction performance of *TM_g_* by Formulae (2–5) in Section 3.2. For each *TM_g_* ∈ {*TM*_1_, *TM*_2_,…, *TM_n_*}, we can calculate its feature extraction performance *FEG_g_* by this way. So, we can get the feature extraction performance of all the models as {*FEG*_1_, *FEG*_2_,…, *FEG_n_*}.

Step 4. Since we have obtained the feature extraction performance of every model, we can calculate the ensemble learning weight of each model by using Formula (6). Then, we can get the ensemble learning weight of all the models as {*w*_1_, *w*_2_,…, *w_n_*}.

Step 5. Once we want to recognize a new crop disease image, we need to input it into the trained models one by one. Then, we can get the softmax function output of each model. After this step, we can get *n* softmax function output. We use *sf_i_* to represent the softmax function output of *TM_i_* as follows:


(8) $$sf_{i}=\left\{s_{1},\;s_{2},\;\ldots ,s_{k}\right\}.$$

In Formula (8), si means that from the perspective of *TM_i_*, the possibility of the new image belonging to *category_j_* is $s_{i}$ .

Step 6. After getting the softmax function output of each trained model, we need to calculate the final softmax function output by Formula (7). Then, we can get the final softmax function output $sf_{final}$ .

Step 7. After completing the previous step, we can have the final softmax function output as follows:


(9) $$sf_{final}=\left\{sc_{1},sc_{2},\ldots ,sc_{k}\right\}.$$

In Formula (9), $sc_{j}$ means the final possibility of the new image belonging to *category_j_*. So, we will generally choose the category that has the maximum possibility value as the final recognition result.

## 4 Experiments

To verify the effectiveness of ELCDR, our experiments were performed on a dataset that includes four kinds of crops. We will mainly address three research questions (RQ) as follows:

1) RQ 1: Can ELCDR achieve better crop disease recognition performance than single model methods?
2) RQ 2: Can our weighting strategy achieve better crop disease recognition performance than the voting and average weighting strategies?
3) RQ 3: Is our feature extraction performance metric effective?

### 4.1 Dataset

**Figure 6:**
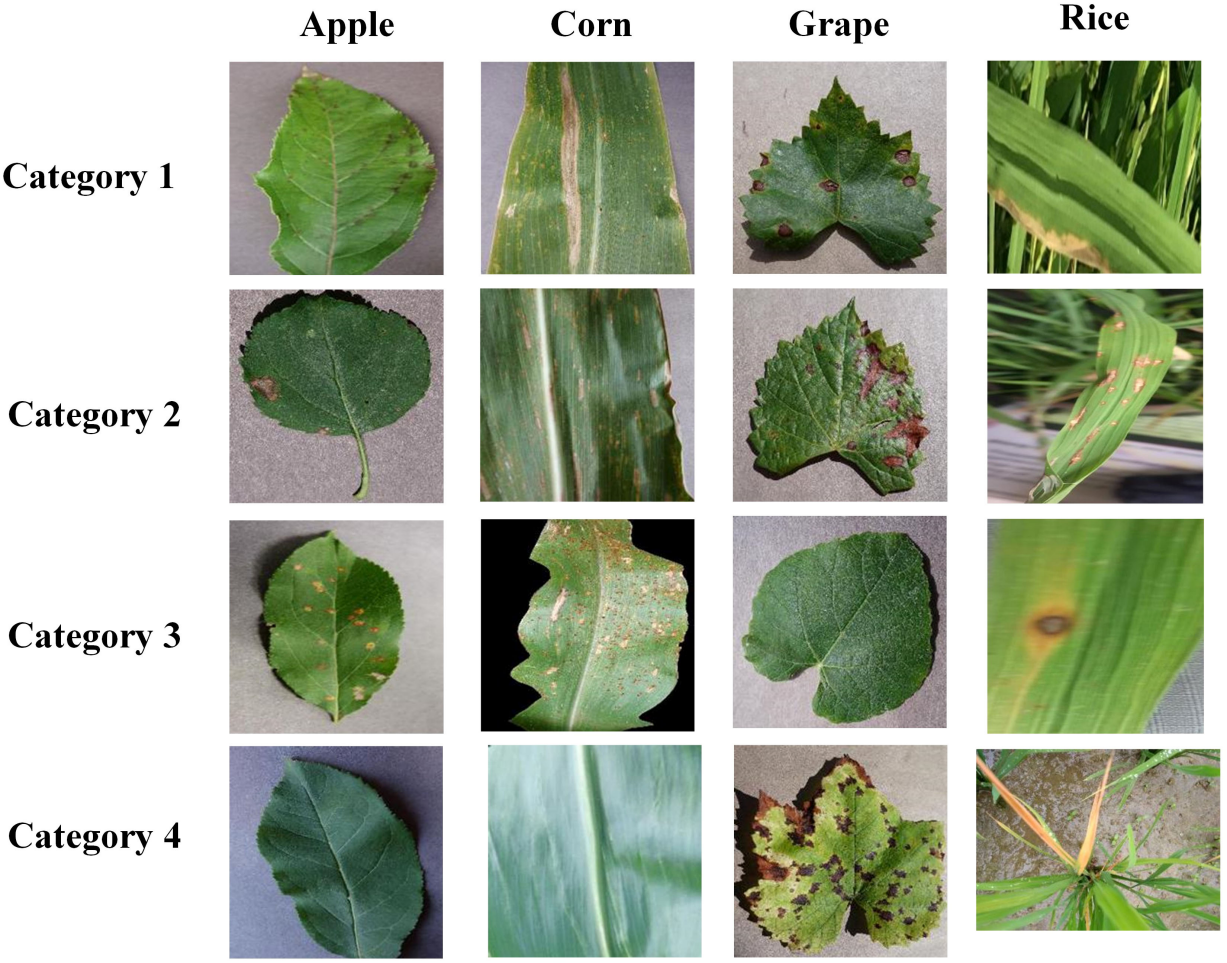
Samples of the dataset.

As shown in **Table 1**, we have built a dataset which includes four kinds of crops, and the details of the dataset are shown in **Table 1**. In this dataset, each crop category has four image categories. The datasets for apple, corn, and grape were taken from the PlantVillage dataset (Hughes and Salathe, 2015), and the images were captured against a simple background. The rice leaf images were taken from the Sambalpur University’s dataset (Sethy et al., 2020), and these images were captured in a natural environment with complex backgrounds. The images in **Figure 6** are the example images of the dataset. The dataset has been uploaded to the Kaggle website and can be accessed by the following website address: https://www.kaggle.com/datasets/zhangguangchuan/crop-disease-dataset.

**Table 1:** Dataset.

Crop	Number of images	Number of categories	Training:test
Apple	2,000	4	3:2
Corn	2,000	4	
Grape	2,000	4	
Rice	2,000	4	

### 4.2 Performance metrics

We used accuracy, precision, recall, and confusion matrix to measure the performance of crop disease recognition methods in our experiments. These metrics are the most common metrics in the research field of image recognition. For their specific definition, please refer to our previous article (He et al., 2022).

### 4.3 Experiment details

**Figure 7:**
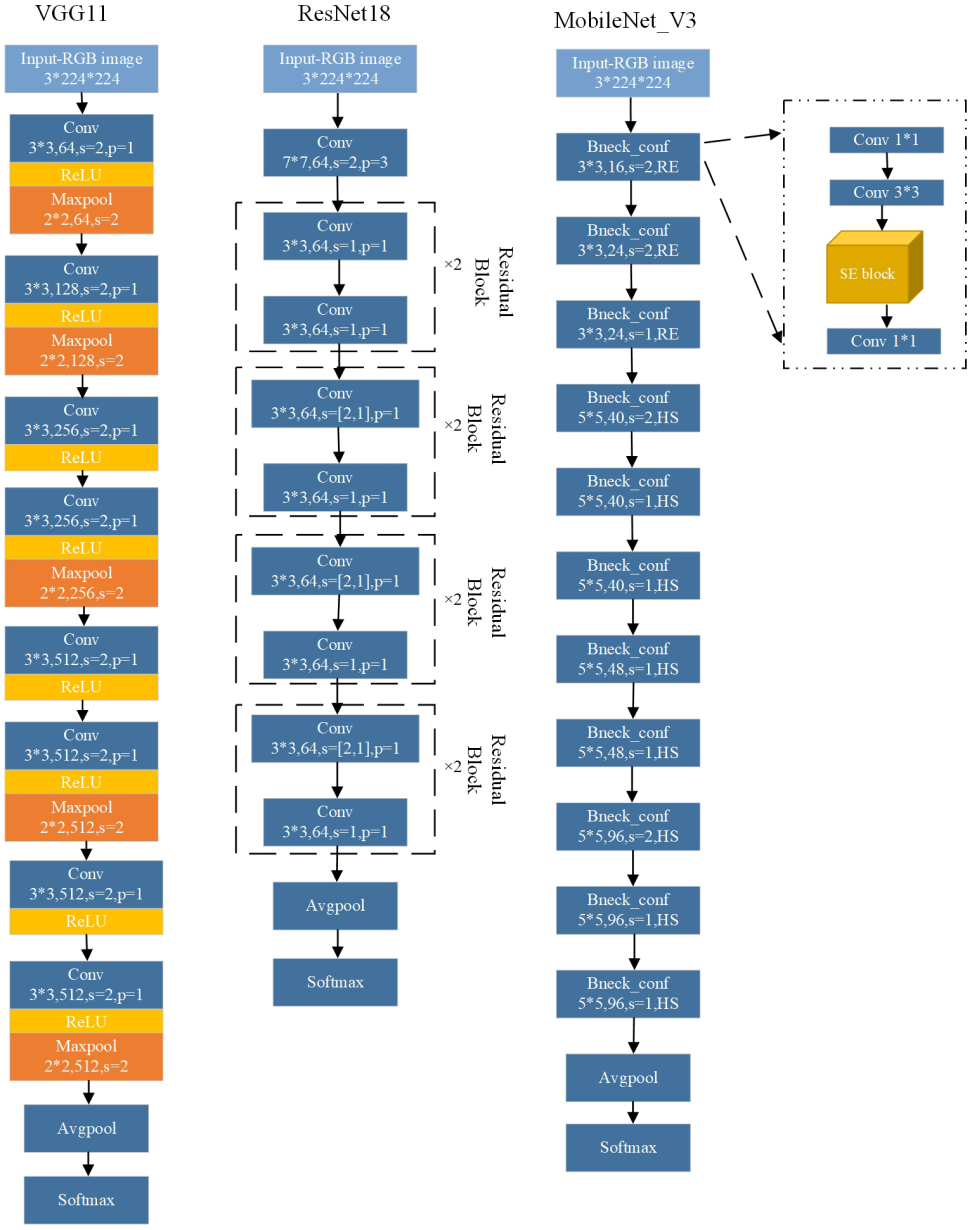
The basic network structure of deep learning models that are deployed in the experiment.

We deployed three DL models in ELCDR, which are widely used in crop disease recognition methods, specifically VGG11, ResNet18, and MobileNet_v3. Their network structure is shown in **Figure 7**. The main idea of the VGG model is to construct a deep network model by reusing simple foundation blocks (Simonyan and Zisserman, 2014). VGG uses small convolutional kernels and pooling layers, with deeper layers and more channels. It hopes to extract more features by increasing the number of channels. VGG11 has 11 parameter layers, consisting of 8 convolutional layers and 3 fully connected layers. ResNet is a deep residual network developed by Microsoft Research Asia (He et al., 2016). It uses residual blocks and residual connections to construct the network, which allows for training deeper networks and avoids gradient vanishing problem. It can achieve better classification performance by continuously increasing the network depth. ResNet18 has 18 parameter layers, including 8 residual blocks. MobileNet_V3 was proposed by Google in 2019 and is the third-generation network of MobileNet (Howard et al., 2019). MobileNet_V3 is a lightweight model and can construct a very small, low latency, and low consumption model by only setting two hyperparameters. MobileNet_V3 mainly consists of 11 bottleneck layers. All of these models are widely applied in crop disease recognition systems and research studies. Thus, we chose them to test the effectiveness of our method in the experiment.

To answer RQ 1, we tested ELCDR using each of the crops from the dataset, and then we compared its performance against that of VGG11, ResNet18, and MobileNet_v3, respectively.

To answer RQ 2, we compared the performance of ELCDR with the voting and average weighting strategies.

To answer RQ 3, we calculated the ensemble learning weight and recognition performance for each model separately to investigate whether our weighting strategy effectively reflects the feature extraction performance of different models.

The hyperparameter settings of the models in the experiment are shown in **Table 2**. The number of epochs was set as 60 in the experiment, as we found that the models’ loss function had basically converged after 50 training epochs in the experiment.

**Table 2:** Hyperparameter settings.

Hyperparameters	VGG11	ResNet18	MobileNet_v3
Learning rate	0.001	0.001	0.001
Batch size	32	32	32
Number of epochs	60	60	60
Optimizer	Adam	Adam	Adam
Loss function	Cross-entropy	Cross-entropy	Cross-entropy

**Figure 8:**
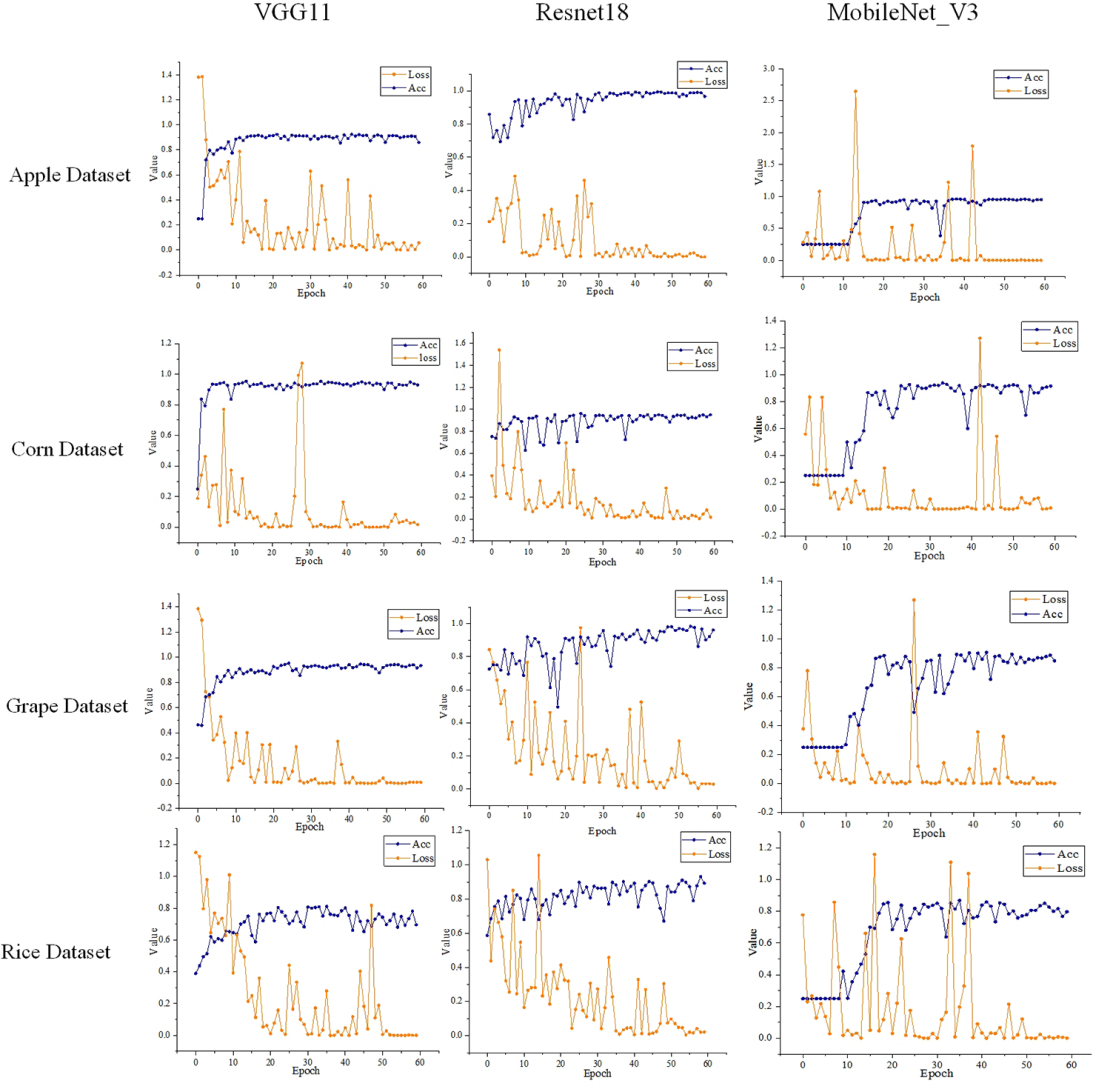
Loss and accuracy of different models on different datasets.

The models’ training loss and accuracy in the training process on different datasets are shown in **Figure 8**. We can find that as the number of training epochs increases, the loss gradually decreases while the accuracy gradually improves. After over 50 training epochs, the models’ loss and accuracy basically no longer show significant changes. During the experiments, we extracted the output from the pooling layer to serve as the feature vectors when calculating the feature extraction performance of a model.

### 4.4 Experiment results

#### 4.4.1 Comparison of recognition performance between ELCDR and different single models

**Figure 9:**
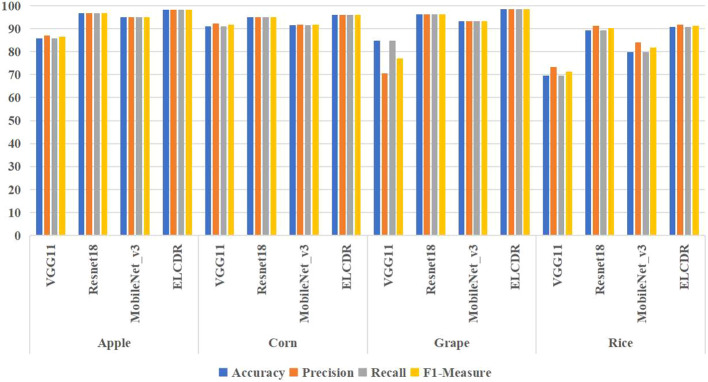
Comparison of the recognition performance between ELCDR and different single models.

In order to answer RQ 1, we compared the recognition performance of ELCDR with VGG11, ResNet18, and MobileNet_v3. The results are shown in **Table 3** and **Figure 9**.

**Table 3:** Comparison of the recognition performance between ELCDR and different single models.

Crop	Model	Accuracy	Precision	Recall	F1 measure
Apple	VGG11	85.88	87.07	85.88	86.47
	ResNet18	96.63	96.70	96.63	96.66
	MobileNet_v3	94.88	94.96	94.88	94.92
	**ELCDR**	**98.13**	**98.14**	**98.13**	**98.13**
Corn	VGG11	91.13	92.26	91.13	91.69
	ResNet18	95.00	95.04	95.00	95.02
	MobileNet_v3	91.50	91.81	91.50	91.65
	**ELCDR**	**95.88**	**96.00**	**95.88**	**95.94**
Grape	VGG11	84.75	70.70	84.75	77.09
	ResNet18	96.13	96.24	96.13	96.18
	MobileNet_v3	93.38	93.34	93.38	93.36
	**ELCDR**	**98.38**	**98.39**	**98.38**	**98.38**
Rice	VGG11	69.50	73.34	69.50	71.37
	ResNet18	89.25	91.21	89.25	90.22
	MobileNet_v3	79.75	84.12	79.75	81.88
	**ELCDR**	**90.75**	**91.86**	**90.75**	**91.30**

Bold values highlight ELCDR performance.

From **Table 3** and **Figure 9**, we can find that the accuracy, precision, recall, and F1 measure of ELCDR are the highest across all crop datasets. The single model that has the best recognition performance is ResNet18, while the single VGG11 model has the worst recognition performance. Compared with ResNet18, ELCDR improves by as much as 1.5 (apple), 0.88 (corn), 2.25 (grape), and 1.5 (rice) percentage points in accuracy in each case. As observed in **Table 3**, ELCDR also has improvements in precision, recall, and F1 measure over the single ResNet18 model.

**Figure 10:**
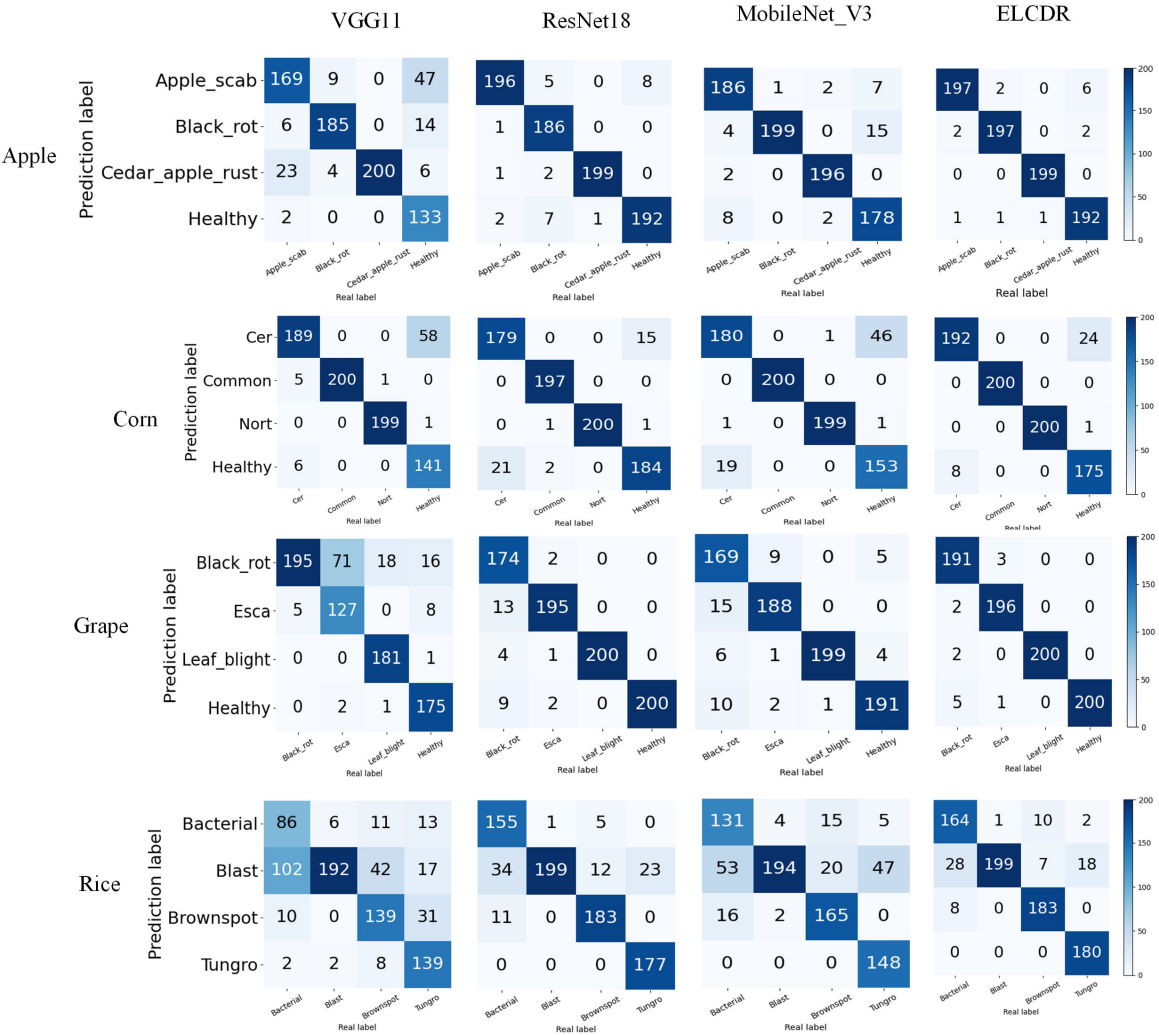
Different models’ confusion matrix on different datasets.

In **Figure 10**, it can be found that ELCDR recognizes the greatest number of correct images on each category of the apple, corn, and rice datasets. In the case of the grape dataset, while ELCDR recognizes a smaller number of correct images than VGG11 in the category of “black rot,” it still recognizes the greatest number of correct images in total.

Based on these results, we can answer research question 1: compared with the single model methods, ELCDR can achieve better crop disease recognition performance. **Table 3** and **Figure 9** show that ELCDR attains higher accuracy, precision, recall, and F1 measure than the methods relying on a single deep learning model. Additionally, **Figure 10** shows that ELCDR can recognize more correct images than the methods that are based on the single deep learning model. These findings have proven that the ensemble learning strategy of ELCDR is effective in achieving a better crop disease recognition performance than the single model methods.

#### 4.4.2 Comparison of the recognition performance between ELCDR and other ensemble learning strategies

**Figure 11:**
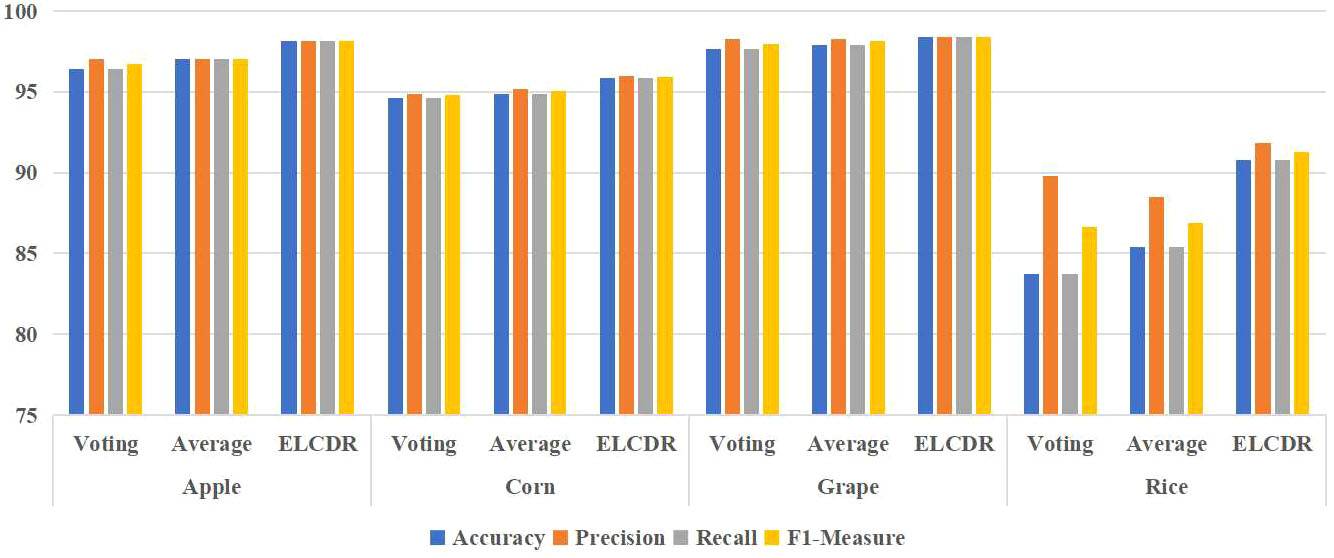
Comparison of the recognition performance between ELCDR and other ensemble learning strategies.


To answer research question 2, we also investigated the performance of the ensemble learning strategy of voting and average weighting. The results are shown in **Table 4** and **Figure 11**. The average weighting strategy calculates the final softmax function output as follows:

**Table 4:** Comparison of the recognition performance between ELCDR and other ensemble learning strategies.

Crop	Method	Accuracy	Precision	Recall	F1 measure
Apple	Voting	96.38	97.03	96.38	96.70
	Average weighting	97.00	97.05	97.00	97.02
	**ELCDR**	**98.13**	**98.14**	**98.13**	**98.13**
Corn	Voting	94.63	94.86	94.63	94.77
	Average weighting	94.88	95.16	94.88	95.02
	**ELCDR**	**95.88**	**96.00**	**95.88**	**95.94**
Grape	Voting	97.63	98.25	97.63	97.94
	Average weighting	97.88	98.24	97.88	98.13
	**ELCDR**	**98.38**	**98.39**	**98.38**	**98.38**
Rice	Voting	83.75	89.79	83.75	86.66
	Average weighting	85.38	88.49	85.38	86.91
	**ELCDR**	**90.75**	**91.86**	**90.75**	**91.30**

Bold values highlight ELCDR performance.

(10) $$sf_{final}=\;\frac{\sum_{i=1}^{n}sf_{i}}{n}.$$

In Formula (10), sfi means the softmax function output of model i, and n means the number of the models. We can see in **Table 4** and **Figure 11**, ELCDR consistently achieves the best recognition performance on the experiment dataset. On the apple, corn, and grape datasets, the average weighting strategy achieves better performance than the voting strategy. On the rice dataset, the voting strategy has better performance than the average weighting strategy. However, ELCDR consistently achieves the best recognition performance regardless of the dataset. Compared with the voting strategy, ELCDR improves by as much as 1.75 (apple), 1.25 (corn), 0.75 (grape), and 7 (rice) percentage points in accuracy in each case. Compared with the average weighting strategy, ELCDR improves by as much as 1.13 (apple), 1 (corn), 0.5 (grape), and 5.37 (rice) percentage points in accuracy in each case. Especially on the rice dataset, the voting strategy and the average weighting strategy both achieve the worse performance than the single ResNet18 model, and the performance improvement of ELCDR is most evident in this case. This might be because the images in the rice dataset have a complex background, making it challenging for the models to extract the efficient feature, while the voting strategy and the average weighting strategy cannot determine which model has extracted the most efficient features. The recognition performance of the voting strategy and the average weighting becomes worse. In contrast, ELCDR can determine which model has extracted the most efficient features and assigns more weight during ensemble learning, consistently achieving better recognition performance. We will further discuss this hypothesis in research question 3.

**Figure 12:**
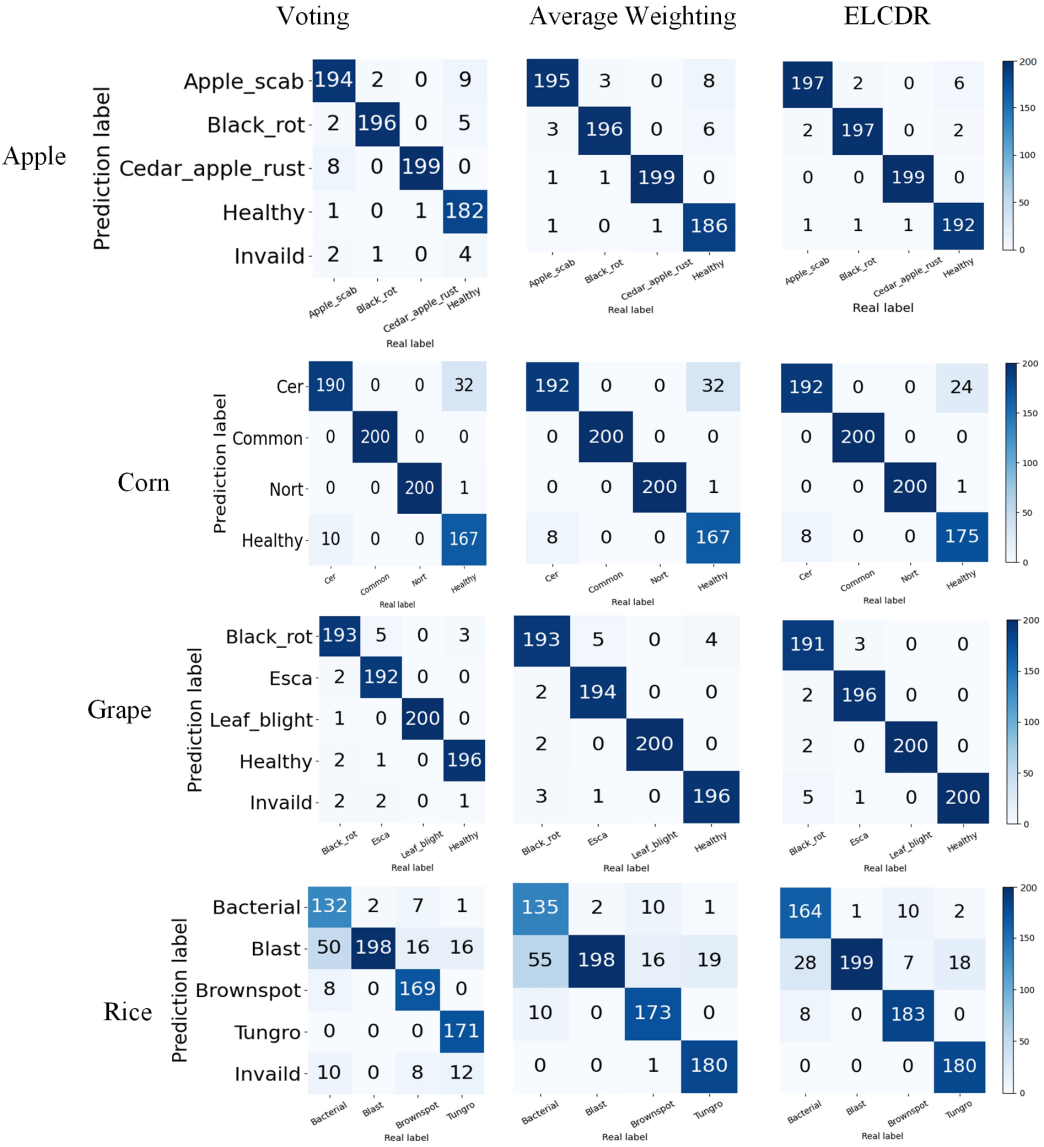
Different ensemble learning strategies’ confusion matrix on different datasets.

In **Figure 12**, we can see that ELCDR consistently recognizes the greatest number of correct images on each dataset. When recognizing an image using the voting strategy, if each model votes for a different category, the voting strategy is considered invalid for that image. We can see that in **Figure 12**, there are consistently some images for which the voting strategy is invalid. This is also the main reason why the voting strategy achieves the worse performance than the other ensemble learning strategies.

By this, we can answer research question 2: the weighting strategy of ELCDR achieves better recognition performance than the voting strategy and average weighting strategy. **Table 4** and **Figure 11** show that ELCDR can achieve better accuracy, precision, recall, and F1 measure than the voting strategy and average weighting strategy. **Figure 12** shows that ELCDR can recognize more correct images than the voting strategy and average weighting strategy. These results have proven that the ensemble learning strategy of ELCDR is more effective than the voting strategy and average weighting strategy.

#### 4.4.3 Comparison of the ensemble learning weights of different models

**Figure 13:**
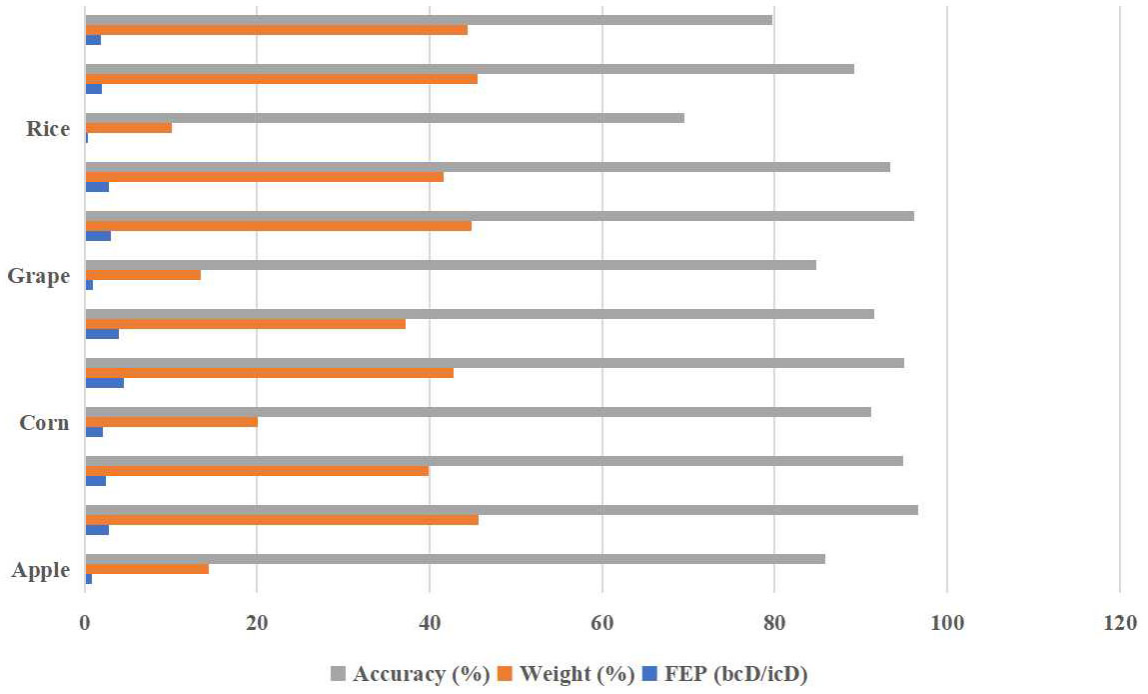
Comparison of the ensemble learning weights of different models.

In order to answer research question 3, we calculated the in-category distance (icD), between-categories distance (bcD), feature extraction performance (FEP), weight, and accuracy performance for each model integrated into ELCDR. The results are shown in **Table 5** and **Figure 13**. We can find that the ResNet18 model consistently achieved the best accuracy performance on each of the datasets, resulting in the highest FEP and ensemble learning weight. Conversely, the VGG11 model consistently demonstrated the worst accuracy performance on each of the datasets, resulting in the lowest FEP and ensemble learning weight. So, the FEP and weight distribution of the models in ELCDR are consistent with their recognition performance. It means that the model that has better feature extraction and recognition performance receives greater weight during ensemble learning with ELCDR, while those with lower performance receive lower weight.

**Table 5:** Comparison of the ensemble learning weights of different models.

Crop	Model	icD	bcD	FEP (bcD/icD)	Weight (%)	Accuracy (%)
Apple	VGG11	2.051201	1.807103	0.880997	14.371	85.88
	ResNet18	8.251477	23.11970	2.801886	**45.706**	**96.63**
	MobileNet_v3	6.055690	14.82056	2.447378	39.923	94.88
Corn	VGG11	1.335760	2.818918	2.110348	20.023	91.13
	ResNet18	4.639691	20.89895	4.504383	**42.738**	**95.00**
	MobileNet_v3	4.316191	16.94026	3.924816	37.239	91.50
Grape	VGG11	1.756650	1.635504	0.931035	13.523	84.75
	ResNet18	6.790820	20.96166	3.086765	**44.833**	**96.13**
	MobileNet_v3	3.948179	11.32035	2.867232	41.644	93.38
Rice	VGG11	2.27075	0.979885	0.431525	10.061	69.50
	ResNet18	11.52429	22.49176	1.951684	**45.504**	**89.25**
	MobileNet_v3	8.528134	16.25342	1.905859	44.435	79.75

Bold values highlight ELCDR performance.

By this, we can answer research question 3 that the feature extraction performance metric of ELCDR is effective. We can use it to measure the model’s feature extraction performance and calculate ensemble learning weight efficiently.

**Figure 14:**
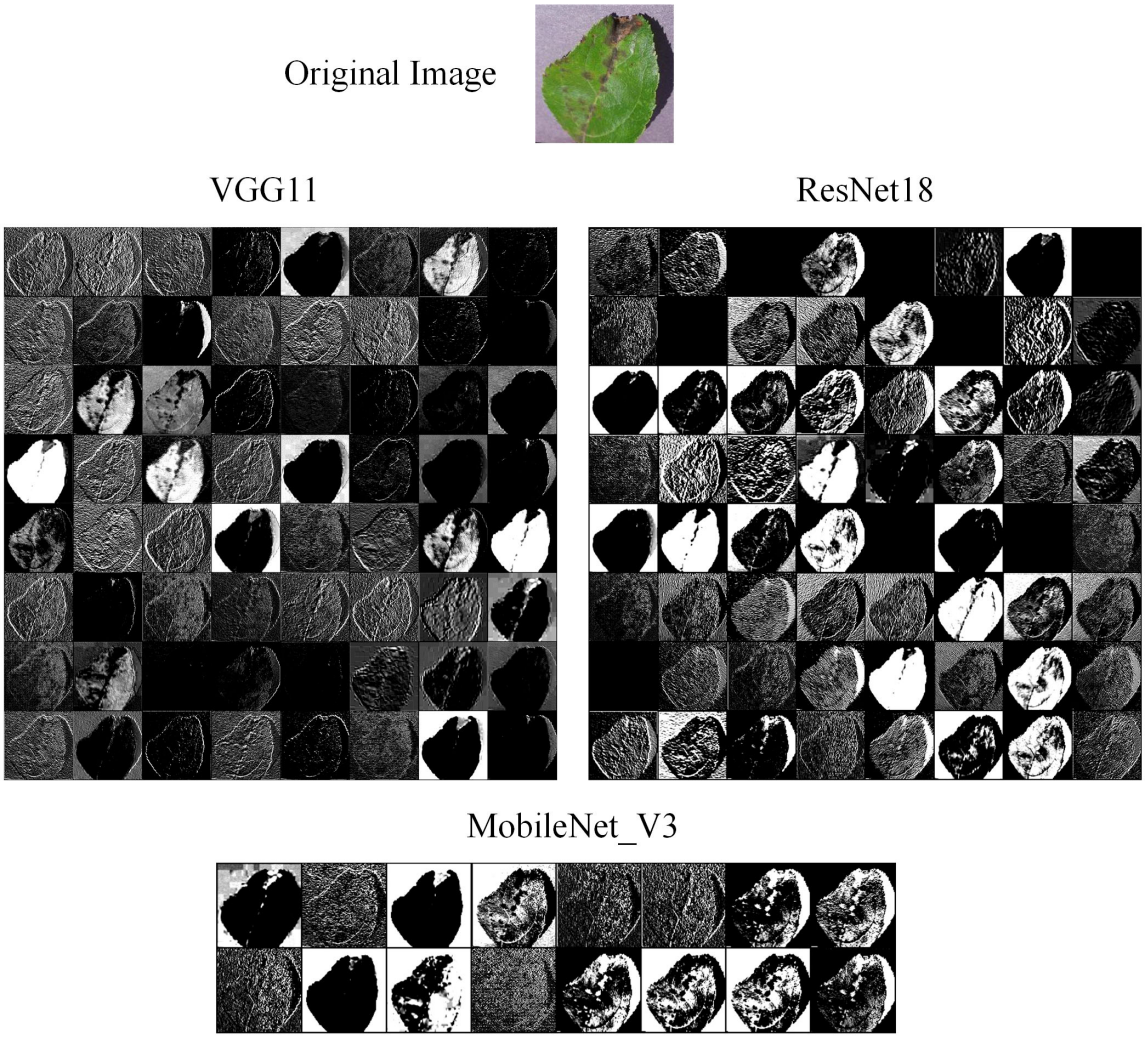
The feature map comparison of the different models.

We also compared the feature maps of different models in the experiment. The original image was input into VGG11, ResNet18, and MobileNet_V3, respectively. Then, we extracted the feature maps from their convolutional layer. The feature maps are shown in **Figure 14**. We can find that the feature map of ResNet18 has the most texture detail features and lesion features, while the feature map of VGG11 is the blurriest. This means that ResNet18 has extracted the most effective features, and VGG11 has extracted the least. MobileNet_V3 is a lightweight model, so it cannot extract as many effective features as ResNet18. However, we also can find that the feature map of MobileNet_V3 has more texture detail features and lesion features than the VGG11. Therefore, we can suggest that different models have different feature extraction performance, which is the main reason why different weights are assigned to these models in ensemble learning.

### 4.5 Discussion

In this section, we conducted experiments to assess the crop leaf recognition performance of ELCDR. The experimental results show that ELCDR can achieve better recognition performance than the methods that are based on a single model. It can also achieve better recognition performance than the traditional ensemble learning methods that rely on the voting or average weighting strategies. This is because we applied a novel weight calculation method, which can measure different models’ feature extraction performance through the distribution of feature vectors. With this weight calculation method, we assign more weight to the models with better recognition performance when conducting ensemble learning for crop leaf disease recognition. Otherwise, the models with poorer performance receive less weight. All of the experimental results verify the effectiveness of the ELCDR as proposed in this paper.

## 5 Conclusions

Compared with the crop leaf disease recognition methods based on single models, ensemble learning methods have the advantage of integrating multiple single learning models to get more accurate, stable, and robust results. This advantage stems from the fact that different models can extract image features from various perspectives. Ensemble learning can combine these features effectively to get a more powerful integrated model.

Traditional ensemble learning methods generally use the voting or average weight strategies, treating all integrated models as equally important. However, different models may have different feature extraction ability. When using ensemble learning, it is essential to assign more weight to the models that have better feature extraction ability and less weight to those that have a weaker feature extraction ability. To solve this problem, we have introduced a novel ensemble learning method for crop leaf disease recognition, named ELCDR. This approach measures each model’s feature extraction ability by calculating the model’s feature vector distribution and calculates the ensemble learning weight for each model based on the model’s feature extraction ability. Through this approach, ELCDR can integrate more effective features from different models, to obtain more accurate, stable, and robust crop leaf disease recognition results. In order to verify the recognition performance of ELCDR, we compared its performance with the recognition methods which are based on a single model, voting strategy, and average weighting strategy in the experiments. The experimental results clearly demonstrate that ELCDR can achieve better accuracy, recall, precision, and F1 measure performance than the recognition methods which are based on a single model, voting strategy, and average weighting strategy. Compared with the VGG11 model, ELCDR improves by as much as 12.25 (apple), 4.75 (corn), 13.63 (grape), and 21.25 (rice) percentage points in accuracy in each case. Compared with the ResNet18 model, ELCDR improves by as much as 1.5 (apple), 0.88 (corn), 2.25 (grape), and 1.5 (rice) percentage points in accuracy in each case. Compared with the MobileNet_V3 model, ELCDR improves by as much as 3.25 (apple), 4.38 (corn), 5 (grape), and 11 (rice) percentage points in accuracy in each case. Compared with the voting strategy, ELCDR improves by as much as 1.75 (apple), 1.25 (corn), 0.75 (grape), and 7 (rice) percentage points in accuracy in each case. Compared with the average weighting strategy, ELCDR improves by as much as 1.13 (apple), 1 (corn), 0.5 (grape), and 5.37 (rice) percentage points in accuracy in each case. These experimental results validate that ELCDR consistently has better recognition performance than the methods that are based on a single model or traditional voting strategy.

We have successfully verified the effectiveness of our proposed feature extraction ability metric in the experiments. However, our new method currently only completes the recognition task in a small range of scenarios. We still face some challenges as follows:

1) The effectiveness of ELCDR on more complex datasets, which may involve a greater variety of crops and harsh environmental conditions, still needs further verification.
2) It remains to be determined how the number of integrated models in ELCDR impacts the recognition performance.
3) Identifying the optimal combination of models to achieve the best recognition performance for ELCDR is another area of potential research.

In the future, we aim to compare the potential benefits and limitations of the existing crop leaf disease recognition methods and explore a robust and accurate crop leaf disease recognition segmentation method.

## Data availability statement

The raw data supporting the conclusions of this article will be made available by the authors, without undue reservation.

## Author contributions

YH: Conceptualization, Writing – original draft, Formal analysis, Funding acquisition, Investigation, Methodology, Project administration, Resources, Software. GZ: Validation, Visualization, Writing – review & editing. QG: Conceptualization, Data curation, Supervision, Writing – review & editing.
